# Regulation of transforming growth factor-beta1 (TGF-β1)-induced pro-fibrotic activities by circadian clock gene *BMAL1*

**DOI:** 10.1186/s12931-016-0320-0

**Published:** 2016-01-12

**Authors:** Chunmin Dong, Rafael Gongora, Meredith L. Sosulski, Fayong Luo, Cecilia G. Sanchez

**Affiliations:** Department of Medicine, Section of Pulmonary Disease and Critical Care, Tulane University School of Medicine, New Orleans, LA USA

**Keywords:** BMAL1, TGF-beta, EMT, Fibroblast to myofibroblast differentiation, Pulmonary fibrosis

## Abstract

**Background:**

BMAL1 is a transcriptional activator of the molecular clock feedback network. Besides its role in generating circadian rhythms, it has also been shown to be involved in the modulation of cell proliferation, autophagy and cancer cell invasion. However, the role of BMAL1 in pulmonary fibrogenesis is still largely unknown. In this study, we investigated the crosstalk between BMAL1 and the signaling transduction and cellular activities of TGF-β1, a key player in lung fibrogenesis.

**Methods:**

Lungs from wild type and TGF-β1-adenovirus-infected mice were harvested and homogenized for isolation of RNA and protein. RT-PCR and Western Blotting were employed to measure the expression level of clock genes and TGF-β1-induced downstream target genes. siRNA against human BMAL1 gene was transfected by using lipofectamine RNAiMAX to knockdown the endogenous BMAL1 in both lung epithelial cells and fibroblasts.

**Results:**

Our results showed that TGF-β1 is able to up-regulate BMAL1 expression in both lung epithelial cells and normal lung fibroblasts. In animal models of pulmonary fibrosis, BMAL1 expression was also significantly higher in adenovirus-TGF-β1-infected mice than in the control group. Interestingly, BMAL1 was mostly found in a deacetylated form in the presence of TGF-β1. Importantly, siRNA–mediated knockdown of BMAL1 significantly attenuated the canonical TGF-β1 signaling pathway and altered TGF-β1-induced epithelial-mesenchymal transition and MMP9 production in lung epithelial cells. In addition, BMAL1 knockdown inhibited the fibroblast to myofibroblast differentiation of normal human lung fibroblasts.

**Conclusions:**

Our results indicate that activation of TGF-β1 promotes the transcriptional induction of BMAL1. Furthermore, BMAL1 is required for the TGF-β1-induced signaling transduction and pro-fibrotic activities in the lung.

## Background

Idiopathic pulmonary fibrosis is a chronic, progressive, irreversible, and age-related lung disease that has no known cause and few treatment options. This disease was once thought to be a chronic inflammatory process, but current evidence indicates that the fibrotic response is driven by abnormally activated alveolar epithelial cells [1–3]. The injured epithelial cells are the primary source of mediators for the development of pulmonary fibrosis, producing a number of growth factors and cytokines, including platelet-derived growth factor, transforming growth factor β (TGF-β), tumor necrosis factor α (TNFα), endothelin-1 and connective tissue growth factor. Among them, TGF-β1 plays a pivotal role in the development of lung fibrosis. It stimulates the fibroblast to myofibroblast differentiation (FMD), the epithelial to mesenchymal transition (EMT) and the production of matrix metalloproteinases (MMPs) to promote the formation of the fibroblast and myofibroblast foci. These foci produce excessive amounts of extracellular matrix, like collagen, resulting in scarring and destruction of the lung architecture [4, 5]. As a matter of fact, TGF-β signaling is consistently found to be upregulated in human pulmonary fibrosis and several experimental lung fibrotic diseases [6–8]. Transduction of active TGF-β1 gene expression induces fibrogenesis in the lung [9, 10] and blockage of the bioactivity of TGF-β1 inhibits matrix production, and represses the progress of lung fibrosis [11, 12].

TGF-β1 is secreted in a latent form that must be activated by cleavage for function. It binds to its heterodimeric receptor on the cell membrane of target cells to activate the receptor’s serine/threonine kinase activity. The activated receptor recruits and phosphorylates the R-Smad protein, Smad2/3, which then forms a complex with the Co-Smad, Smad4. The complexes then translocate into the nucleus to regulate transcription of the target genes in cooperation with other co-factors [13, 14]. Besides the canonical Smad2/3 pathway, TGF-β1 can also activate mitogen-activating protein kinases (MAPKs) (ERK, p38 and JNK), phosphatidylinositol 3 kinase/Akt and small GTPases in a cell-specific manner [15–17].

Circadian rhythms occur around a 24-h oscillation in behavior and physiology associated with the solar day. These daily rhythms are driven by a network of transcriptional-translational feedback loops that exist in essentially all tissue and cell types of the organism [18]. Brain and muscle Arnt-like protein 1 (BMAL1), is one of the core elements of these feedback loops. BMAL1 belongs to the family of bHLH-PAS domain transcription factors. It binds to canonical E-box motifs (CACGTG) as a heterodimer with circadian locomotor output cycles kaput (CLOCK) or neuronal PAS domain protein 2 (NPAS2) on its target promoters to activate transcription. They include the Pers (Period1, 2 and 3) and Crys (Chryptochrome 1 and 2), whose protein products accumulate in the cytoplasm and ultimately translocate to the nucleus. Once in the nucleus, Pers and Crys associate with BMAL1/CLOCK complex and suppress gene activation. Meanwhile, this transcriptional negative feedback loop is the subject of an extensive posttranslational control through which oscillations are critically sustained and adjusted to a period of ~24 h [19, 20].

BMAL1 is an essential component of the molecular feedback network. Inactivation of *BMAL1* in mice results in a complete loss of circadian rhythm under constant dark conditions [21]. *BMAL1−/−* mice is characterized by significantly reduced lifespan compared to their wild-type littermates and displayed various symptoms of premature aging as early as 5 months of age with inefficient epidermal self-renewal, less subcutaneous fat, organ shrinkage. This early aging phenotype correlates with increased levels of reactive oxygen species in several tissues [22]. Meanwhile, *BMAL1* knockout cells express lower amounts of the miRNA-23b/-27b/-24-1 cluster, which targets transforming growth factor beta receptor 2 and Smad proteins [23]. In addition, BMAL1 has also been identified as a candidate gene for susceptibility to hypertension and Type II diabetes [24].

Whereas BMAL1 has been reported to regulate the expression of genes involved in different physiological and pathological activities, the expression of BMAL1 itself is under the influence of internal and external environmental events. Rev-Erbα and retinoic acid receptor-related orphan receptor alpha (RORα), two downstream targets of BMAL1, have been recognized to compete the ROR response elements at the promoter region of *BMAL1* to regulate the level of BMAL1 [25, 26]. In addition, the expression of BMAL1 has also been reported to be regulated by glucocorticoid, glucagon, melatonin, and TNFα in different systems [27–30]. However, TGF-β2 has been found to profoundly inhibit the expression of several circadian clock genes, such as *Period* genes, *rev-erbα,* and the clock-controlled genes *Dbp* and *Tef,* without influencing the level of *BMAL1* in NIH3T3 fibroblasts and HT22 neurons [31]. In this study, we assessed the role of TGF-β1 in the expression of clock genes both in vivo and in vitro. We found that overexpression of TGF-β1 in mouse lungs altered the expression profile of circadian clock genes and elevated the expression level of BMAL1 in lung fibroblasts and epithelial cells.

The lung has been demonstrated to exhibit robust circadian rhythm in culture. Using the Per2: luc transgenic mice, Dr. Gibbs’ group recorded bioluminescence circadian oscillation in lung slices [32]. Furthermore, another group revealed that a number of genes that are involved in regulation, maintenance and repair of the lung tissues show oscillation in their expression levels [33]. Recently, BMAL1 has been reported to be an important mediator of inflammatory response. Targeted deletion of *BMAL1* in lung epithelium augments inflammation in response to tobacco/cigarette smoke [34]. Targeted loss of *BMAL1* in bronchiolar cells induces an exaggerated inflammatory response to lipopolysaccharide and impaired host response to streptococcus pneumonia infection [27]. However, it is still largely unknown whether clock genes, especially *BMAL1*, are involved in the development of lung fibrosis. To answer this question, the current study investigated the relationship between BMAL1 and TGF-β1-induced signaling transduction and cellular activities, which play a pivotal role in lung fibrosis. Briefly, we found that siRNA-mediated knockdown of *BMAL1* significantly attenuated TGF-β1-induced signaling transduction cascades and physiological functions in both lung fibroblasts and epithelial cells, suggesting the BMAL1 is required for the proper conduction of TGF-β1 signals and the fibrogenesis in the lung.

## Methods

### Antibodies and reagents

Recombinant human TGF-β1 and TNFα were purchased from R&D Systems (Minneapolis, MN). Lithium chloride (LiCl) was purchased from Sigma-Aldrich (St. Louis, MO) and SB216763 from Selleckchem.com (Houston, TX). Antibodies to E-Cadherin (E-cad), β-actin, GAPDH, Smad3, phosphor-Smad3 (Ser423/425), Akt, phospho-Akt (Ser473), GSK3β, phospho-GSK3β (Ser9), peroxidase-conjugated goat anti-mouse and goat anti-rabbit secondary antibodies were purchased from Cell Signaling (Danvers, MA). Antibody to alpha-smooth muscle actin (α-SMA) was purchased from Sigma Aldrich (St. Louis, MO). Antibodies to fibronectin extra domain A (FN-EDA) and type-1 collagen (col-1) were purchased from Abcam (Cambridge, MA). Antibody to BMAL1 was purchased from Novus (Littleton CO). Acetyl-BMAL1 (K538) antibodies were from EMD Millipore (Billerica, MA) and Ameritech Biomedicines (Houston, TX). Plasminogen activator inhibitor type 1 (PAI1) antibody was obtained from Peprotech (Rocky Hill, NJ).

### Cell culture and transfection

The immortalized human small airway epithelial cell line HPL1D was provided by Dr. Takahashi and maintained in Ham’s F12 medium supplemented with 1 % fetal bovine serum (FBS), 5 μg/ml insulin, 5 μg/ml human transferrin, 10^−7^ M hydrocortisone, and 2 × 10^−10^ M thyronine at 37 °C [35]. The human lung adenocarcinoma A549 cell line was obtained from American Type Culture Collection (ATCC, Manassas, VA) and maintained in Dulbecco’s minimal essential medium (DMEM) (Invitrogen, Eugene‚ OR) supplemented with 10 % FBS, 10 units/ml penicillin, and 10 μg/ml streptomycin (Invitrogen). Normal human lung fibroblasts (NHLFs) were purchased from Lonza (Allendale, NJ) and maintained in FGM-2 medium following manufacturer’s instructions.

siRNAs against human *BMAL1*(siBMAL1), a mixture of 4 siRNA oligos, and all-star control siRNA (siCtrl) were purchased from Qiagen (Valencia, CA). Cells were seeded onto 6-well cell culture plates and 20 μM of siRNA were transfected by using lipofectamineRNAiMAX (Invitrogen). Cells were treated with TGF-β1 and TNFα one day’s later. During treatment with recombinant human TGF-β1, A549 cells were cultured in DMEM plus 0.5 % FBS and NHLF were cultured in FBM plus 0.2 % bovine serum albumin (BSA). Cells were harvested 48 or 72 h following transfection for isolating RNA or proteins.

### Animals and TGF-β1 adenovirus exposure

C57BL/6 mice (Charles River) were maintained in 12-h light, 12-h dark cycles with free access to food and water. All procedures were performed in accordance with protocols approved by the Institutional Animal Care and Use Committee of Tulane University. TGF-β1 adenovirus (advTGF-β1) or control green fluorescent protein virus (advGFP) were gifts from Dr. Gilbert F. Morris (Tulane University). 3 × 10^8^ plaque-forming units (PFU) virus in 50-μl sterile saline were given to mice intratracheally. Mice were sacrificed on day 7 after instillation. Left lungs were fixed for trichrome and immunostaining staining and right lungs were harvested and homogenized in liquid nitrogen for real time quantitative PCR and western blot analysis.

### Histology and immunostaining

Masson’s trichrome staining and analysis was performed as previously described [36]. VECTASTAIN Elite ABC Kit, Bloxal, Avidin/Biotin Blocking Kit, DAB Peroxidase Substrate Kit, and Vector Hematoxylin were obtained from Vector Laboratories (Burlingame, CA, USA). Buffer components Triton X-100 (TX-100) and Tween-20 were purchased from Sigma and glycine was purchased from Fisher Scientific (Pittsburgh, PA, USA).

For immunohistochemical analysis, paraffin embedded samples were incubated in BLOXALL™ Blocking solution (Vector Laboratories, Burlingame, CA, USA) for 10 min before proceeding with antigen retrieval. Slides were incubated for 20 min in 0.5 % ammonium chloride and rinsed in PBS then incubated in 0.3 M Glycine for 10 m. Slides were permeabilized (PBS,0.1 % Triton X-100, 0.5 % Tween 20 ) and blocked (PBST, 2 % BSA, 10 % goat serum) for 30 min. The primary antibodies of BMAL1 (Novus) was used at dilutions of 1:200, and incubated for 30 min followed by diluted biotinylated secondary antibody for another 30 min. Slides were then incubated with Vectastain®ABC Reagent and DAB according kit instructions, and the nuclei counterstained with haematoxylin and mounted with Permount mounting medium. For the analysis of the Masson’s Trichrome staining, 5–7 fields per mouse at 20× in three mice analyzed per group. The IHC analysis for BMAL1 was done in 5–8 fields per mouse at 20× in three mouse per group (advGFP/advTGF-β1). Images were captured at the same magnification with similar contrast, using an Olympus BX60 microscope equipped with epifluorescence optics (Olympus, Melville, NY) and a charge-coupled device camera (DP71, Olympus ) with associated software Cell Sens Standard. All numerical values are expressed as mean ± SEM. TIFF/JPEG file images were then exported and analyzed using ImageJ (imagej.nih.gov).

### Quantitative real-time reverse transcription-PCR

Total RNA was isolated with RNeasy Plus mini kit (Qiagen) following the manufacturer’s protocol and the concentration was measured using NanoDrop spectrophotometer from Thermo Scientific (Wilmington, DE). Real-time quantitative reverse transcription-PCR (qRT-PCR) analysis was performed using an iCycler device (Bio-Rad Laboratories, Hercules, CA) and SYBR green supermix (Bio-Rad) according to the manufacturer’s instructions, along with gene-specific primers (Table 1). The specific gene’s cycle threshold (Ct) values were normalized to the housekeeping gene 36B4 and compared with the control group that was assigned a value of 1 to calculate the relative fold change in expression. The results represented three independent experiments.

**Table 1:** Primer sequences for RT-PCR

Gene	Forward primer sequence (5'-3')	Reverse primer sequence (5'-3')
h-α-SMA	GAAGAAGAGGACAGCACT	TCCCATTCCCACCATCAC
h-Collagen-1	CGGAGGAGAGTCAGGAAGG	CACAAGGAACAGAACAGAACA
36B4	CGACCTGGAAGTCCAACTAC	ATCTGCTGCATCTGCTTG
h-E-cad	ATTCTGATTCTGCTGCTCTTG	AGTCCTGGTCCTCTTCTCC
h-PAI1	GGCTGGTGCTGGTGAATGC	AGTGCTGCCGTCTGATTTGTG
h-FN	GGAGAGAGTCAGCCTCTGGTTCAG	AGTCCACTGGGCGCTCAGGCTTGTG
m-BMAL1	TTTGGGCTAGCTGTGGATAG	AAATATCCACATGGGGGACT
m-Per1	CATCTCAGCGGAGTTCTCAT	CTGGTAGATGGGTTGTCCTG
m-Per2	CTGACGCACACAAAGAACTG	GTAGGAAGGCACATCCACAC
m-Per3	GAGGCACACTAAGCCCAGTA	CGCGAAGGTATCTGTGTTCT
m-Npas2	TGGGAATCCACGATAAGAAA	GGAATTCTGGAATCGTTGTG
m-Nr1d1	GCTTCTCTCAGTTCCCACAA	GGCATTGAAGTTACCAGGTG
m-Nr1d2	TGCAGAATGACACCTTAGCA	GGCATGCTCTCAGATGAGTT
m-DBP	ACCTGACTTCCTCCTTGTCC	CCCGGGTTCTCAAGATTTAT
h-BMAL1	GGAAAAATAGGCCGAATGAT	TGAGCCTGGCCTGATAGTAG
h-MMP9	TGACAGCGACAAGAAGTG	CAGTGAAGCGGTACATAGG

### Western blot

Cells were lysed by 1 × RIPA buffer (Cell Signaling Technology) containing 1 mM PMSF (Sigma Aldrich). An equal amount of proteins were separated by a 4–12 % Novex Tris-Glycine SDS polyacrylamide gel (Invitrogen) followed by transfer onto polyvinylidene difluoride membranes (0.45 μm, Invitrogen). Membranes were blocked in 5 % non-fat milk in Tris-buffered saline containing 0.05 % Tween 20 (TBST) buffer for 1 h at room temperature and then incubated with the appropriate primary antibodies overnight at 4 °C. After washing 3 × 10 min with TBST, blots were incubated with peroxidase-conjugated goat anti-mouse or goat anti-rabbit secondary antibodies for 1 h at room temperature. An ECL plus western blotting detection kit (GE Healthcare, Buckinghamshire, UK) was used for generating chemiluminescent signals. All western blots were repeated at three times and densitometry analysis was performed using National Institutes of Health (NIH) ImageJ software and normalized with β-actin.

### Immunofluorescence

A549 cells cultured on 8-well chamber slides were fixed with ice cold methanol for 10 min at −20 °C freezer. Fixed cells were then incubated with phosphate-buffered saline (PBS) containing 0.02 % Tween 20 (PBST) with 10 % goat serum and 5 % BSA for 1 h followed by anti-E-cad antibodies (1:200) in PBST containing 1 % BSA for 1 h at room temperature. After three washes with PBS, the cells were incubated with Alexa 594-conjugated goat anti-rabbit secondary antibody (1:1000, Invitrogen) for 1 h, which was followed by another three washes with PBS. Cells were then stained with DAPI for 10 min at room temperature. After washing by PBS, the chambers were removed and the slides were mounted with ProLong Gold Antifade Mountant (Life Technologies, Grand Island, NY) and covered with cover slips. Cells were examined with an Olympus BX43 microscope (OLYMPUS Corporation, Tokyo, Japan).

### Gelatin zymography

HPL1D cells transiently transfected with siBMAL1 or control siRNA were treated with TNFα (10 ng/ml) and/or TGF-β1 (5 ng/μl). Conditioned medium was collected 48 h after the treatments and processed for gelatin zymography. 20 μl of unconcentrated conditioned medium was used for gelatin zymography using precast Novex gelatin zymogram gels (Invitrogen) as described previously [37].

### Migration assay

HPL1D cells in 6-well plates were transfected with siRNA against *BMAL1* for 24 h and followed by treatment of TGF-β1 at 5 ng/ml for another 24 h. Cells were then seeded in the 24-well transwell non-coated inserts (CORNING, Corning, NY) at a density of 5 × 10^4^ in triplicates. Full F12 media containing 5 % FBS were added in the bottom chambers. Following incubation for 24 h, the cells were stained following the manufacturer’s instructions. The membranes were removed and mounted on microscope slides with immersion oil. The slides were imaged by Aperio ScanScope slide scanner (Leica Biosystems, Buffalo Grove, IL), and the total cell numbers were counted using ImageJ software.

### Statistical analysis

Differences were evaluated using Student’s *t* test or ANOVA, and *p* < 0.05 was considered as statistically significant. Data are expressed as the mean ± S.E.M.

## Results

### TGF-β1 elevates BMAL1 expression both in vitro and in vivo

**Fig. 1:**
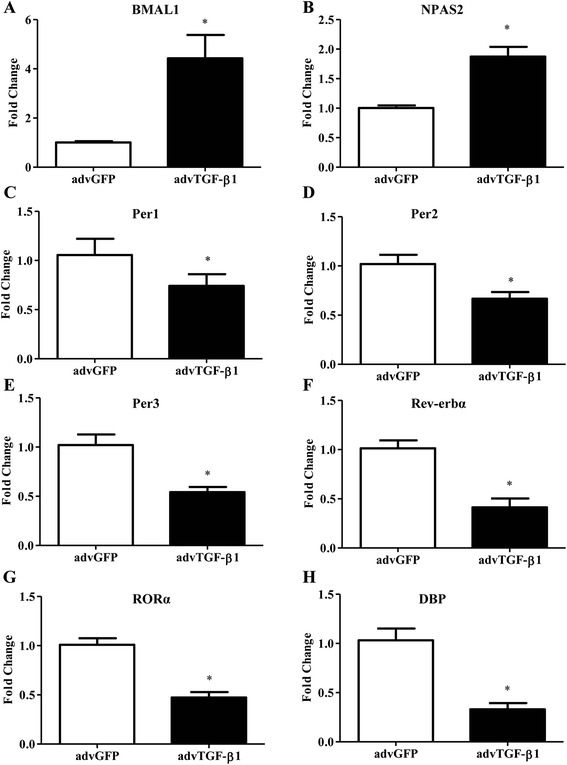
Expression of circadian clock genes in TGF-β1 adenovirus-infected (advTGF-β1) mouse lungs. **a**-**h** C57BL/6 mice were administered 3 × 10^8^ PFU of advTGF-β1 or advGFP by oropharyngeal aspiration. The lung was harvested at day 7 post exposure to advTGF-β1. Real-time qRT-PCR analysis was conducted to investigate the expression of clock genes, as indicated in each panel on the top. * *p* < 0.05 *vs.* advGFP control

In order to delineate the circadian clock gene expression pattern in lung fibrosis, qRT-PCR was employed to investigate the mRNA levels of circadian clock genes in advTGF-β1-induced lung fibrosis model. C57BL/6 mice were exposed to a replication-deficient adenovirus encoding active TGF-β1 (advTGF-β1) or control (advGFP) via oropharyngeal instillation as a model for lung fibrosis (5 mice per group) [10, 36, 38]. Mice were sacrificed 7 days later and lungs were harvested for further analysis. The results of qRT-PCR analyses showed an up-regulation of *BMAL1* and *NPAS2* and down-regulation of *Per1*, *Per2*, *Per3*, *Rev-erbα*, *RORα* and *DBP* in advTGF-β1 mice (Fig. 1). Previously BMAL1 was shown to regulate the transcriptional control of key components of the TGF-β pathway in other tissues [39]. Therefore, we choose to interrogate the role of BMAL1, as a putative regulator of the TGF-β1 signaling pathway during lung fibrogenesis [23].

**Fig. 2:**
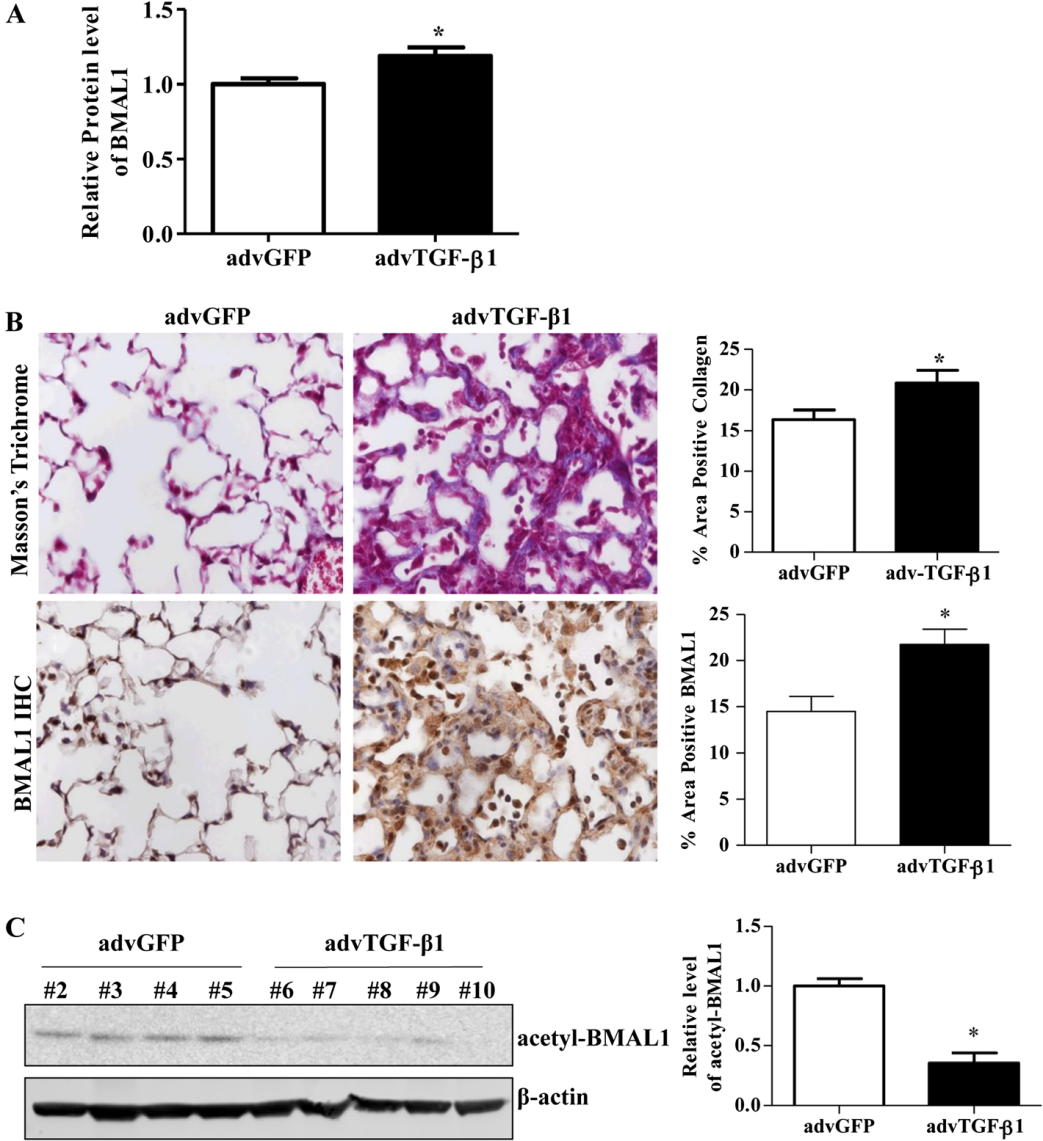
Expression of BMAL1 in advTGF-β1-infected mouse lungs. **a** Densitometric analysis of Western blots of BMAL1 protein expression in the lung homogenate from advTGF-β1-infected mice and Adv-GFP. **b** Representative images and quantification of Immunohistochemistry analysis to quantify BMAL1 expression and Masson’s trichrome staining to evaluate collagen deposition in lungs from advTGF-β1-infected mice versus Adv-GFP. Positive collagen deposition appears blue. Image J quantification of the respective staining is presented. Data represents the mean ± S.E.M., *n* > 5, * *p* < 0.05 *vs.* advGFP control. **c** Western blot analysis of acetyl-BMAL1 in the lung homogenate of advTGF-β1-infected mice. Quantification of BMAL1 and acetyl-BMAL1 levels was done by ImageJ and the densities were normalized by β-actin, * *p* < 0.05 *vs.* advGFP control

In the next set of experiments, the protein expression levels of BMAL1 were investigated and the Densitometry analysis of Western blots showed that BMAL1 was up-regulated in advTGF-β1 group, with a moderate but significant increase of 19 % compared to control group (Fig. 2a). The result was further confirmed by Immunohistochemistry. Briefly, we confirmed a significant tissue remodeling and collagen deposition, measured by trichrome staining, after exposure to advTGF-β1 compared to control. The changes in collagen deposition and alveolar wall thickness were concomitant with an increase in BMAL1 staining (Fig. 2b). The results revealed a significant increase in the expression of BMAL1 in the fibrotic tissue.

It is known that post-translational modification on BMAL1 mediates its activity [40]. To better understand the changes on BMAL1, we evaluated the levels of acetylated BMAL1 (K538), by western-blot from whole lung tissues. The results showed that acetyl-BMAL1 was down-regulated by 65 % in advTGF-β1 groups in comparison with the advGFP control mice (Fig. 2c). The results suggest that TGF-β1 signaling may regulate BMAL1 at multiple levels, including the mRNA and post-translational modifications.

**Fig. 3:**
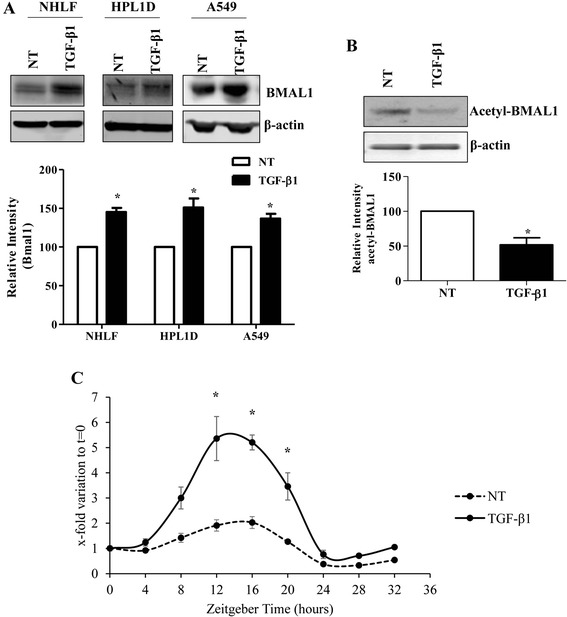
Expression of clock gene BMAL1 in response to TGF-β1 in lung cell lines. **a** Western blot analysis of BMAL1 protein expression in response to TGF-β1 treatment for 24 h in NHLFs, HPL1D, and A549 cells. * *p* < 0.05 vs. corresponding no treatment group (NT). **b** HPL1D cells were treated with TGF-β1 at 5 ng/ml for 24 h and Western blot analysis was performed using antibodies against acetyl-BMAL1 (K538). * *p* < 0.05 vs. NT. **c** HPL1D cells were treated with TGF-β1 at 5 ng/ml after serum-shock by incubating in media containing 50 % horse serum for 2 h. Cells pellets were harvested every 4 h. Real-time qRT-PCR analysis was employed to investigate the expression level of BMAL1. * *p* < 0.05 *vs.* corresponding time points in no treatment (NT) group

To further clarify the effect of TGF-β1 on BMAL1 expression, we investigated the expression level of BMAL1 in response to TGF-β1 in three different lung cell lines, including epithelial cells (normal lung epithelial cell line HPL1D and human lung adenocarcinoma epithelial cell line A549) and normal human lung fibroblasts (NHLFs). Both epithelial cells and fibroblasts are major resources of myofibroblasts in response to TGF-β1 during the development of lung fibrosis. Results from Western blot analysis showed that BMAL1 was up-regulated by TGF-β1 treatment at protein level in all three cell lines investigated (Fig. 3a). We also confirmed that TGF-β1 reduced the level of acetyl-BMAL1 in HPL1D cells (Fig. 3b). At the cellular level, repeated circadian oscillations can be induced by serum shock [41]. To evaluate the effects of TGF-β1 in BMAL1 oscillations, a time-course analysis for BMAL1 by real-time qRT-PCR was performed. The results indicated that BMAL1 mRNA level increased at multiple time points, from zt8 to zt20 following TGF-β1 stimulation in HPL1D cells (Fig. 3c).

### Knockdown of BMAL1 attenuated TGF-β1-induced FMD

**Fig. 4:**
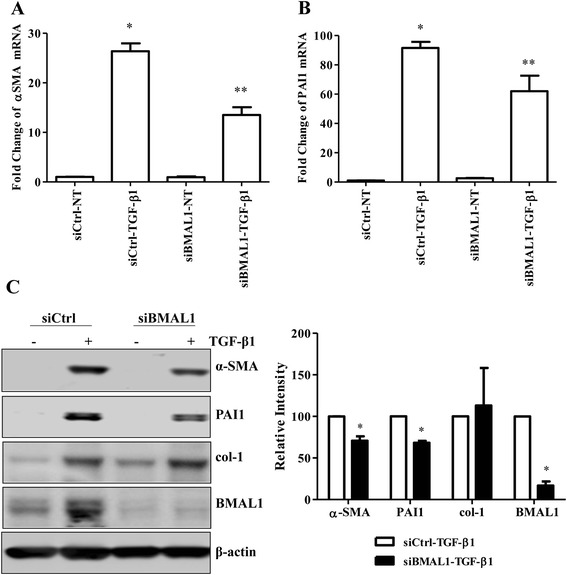
Effect of BMAL1 on TGF-β1-induced FMD. NHLFs were transiently transfected with siBMAL1 or control siRNA (siCtrl) for 48 h. During the last 24 h of transfection, the cells were treated with TGF-β1 at 1 ng/ml. Real-time qRT-PCR analysis was employed to investigate the expression of α-SMA (**a**) and PAI1 (**b**), * *p* < 0.05 *vs.* siCtrl-NT; ** *p* < 0.05 *vs.* siCtrl-TGF-β1. **c** Western blot analysis was carried out to investigate the TGF-β1-induced α-SMA, PAI1 and col-1 expression in response to BMAL1 knockdown. Densitometry analysis by ImageJ is presented. * *p* < 0.05 vs. corresponding control siRNA groups stimulated with TGF-β1 (siCtrl-TGF-β1)

Since TGF-β1 is able to up-regulate BMAL1 expression in the lung, we have interrogated the function of BMAL1 in TGF-β1 induced activities in the lung. For this, we transfected a mixture of four siRNA oligos targeting different sequences of human *BMAL1* gene to knock down the expression of BMAL1 in NHLFs, HPL1D and A549 cells, and the effects of BMAL1 knockdown on the TGF-β1-induced activities were investigated. To eliminate the possibility of nonspecific silencing effects, AllStars Negative Control siRNA, a nonsilencing siRNA that was thoroughly tested and validated with no homology to any known mammalian gene, was included as a negative control. As shown in the Western blot results, the expression of BMAL1 was markedly reduced by siRNA transfection in NHLFs (Fig. 4c). It is well documented that TGF-β1 promotes FMD and matrix deposition, reflected by the up-regulation of α-SMA and col-1 expression, in fibroblasts. When BMAL1 level was down, the TGF-β1-induced α-SMA expression was significantly attenuated, but not the col-1 expression (Fig. 4a, c). Meanwhile, the TGF-β1-induced PAI1 expression was also significantly attenuated by BMAL1 knockdown (Fig. 4b, c).

### Knockdown of BMAL1 attenuated TGF-β1-induced EMT

**Fig. 5:**
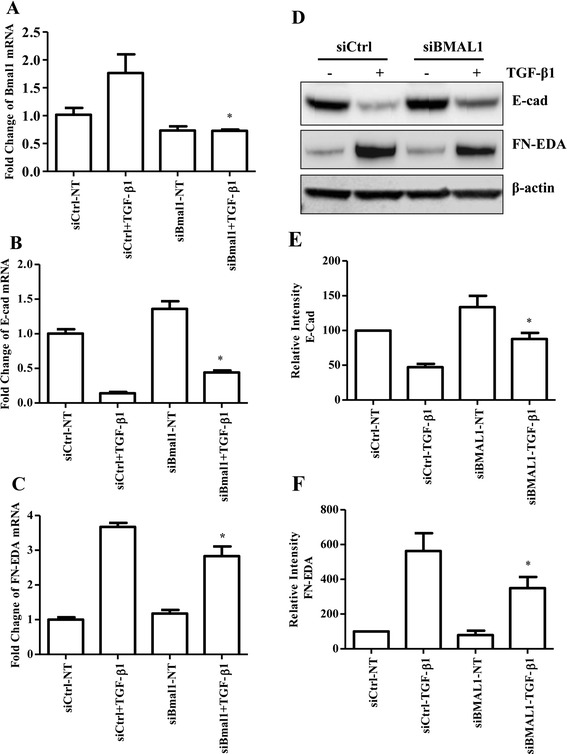
Effect of BMAL1 on TGF-β1-induced EMT of HPL1D lung epithelial cells. HPL1D cells were transiently transfected with siBMAL1 or siCtrl for 48 h. During the last 24 h of transfection, the cells were treated with TGF-β1 at 5 ng/ml. Real-time qRT-PCR analysis was employed to investigate the expression of BMAL1 (**a**), E-cad (**b**) and FN-EDA (**c**). **d** HPL1D cells were transiently transfected with siBMAL1 or siCtrl for 72 h. During the last 48 h of transfection, the cells were treated with TGF-β1 at 5 ng/ml. Western blot analysis was carried out to investigate the E-cad and FN-EDA expression. **e & f** Densitometry analysis of Western blots in (**d**). * *p* < 0.05 vs. corresponding control siRNA groups stimulated with TGF-β1 (siCtrl-TGF-β1)

**Fig. 6:**
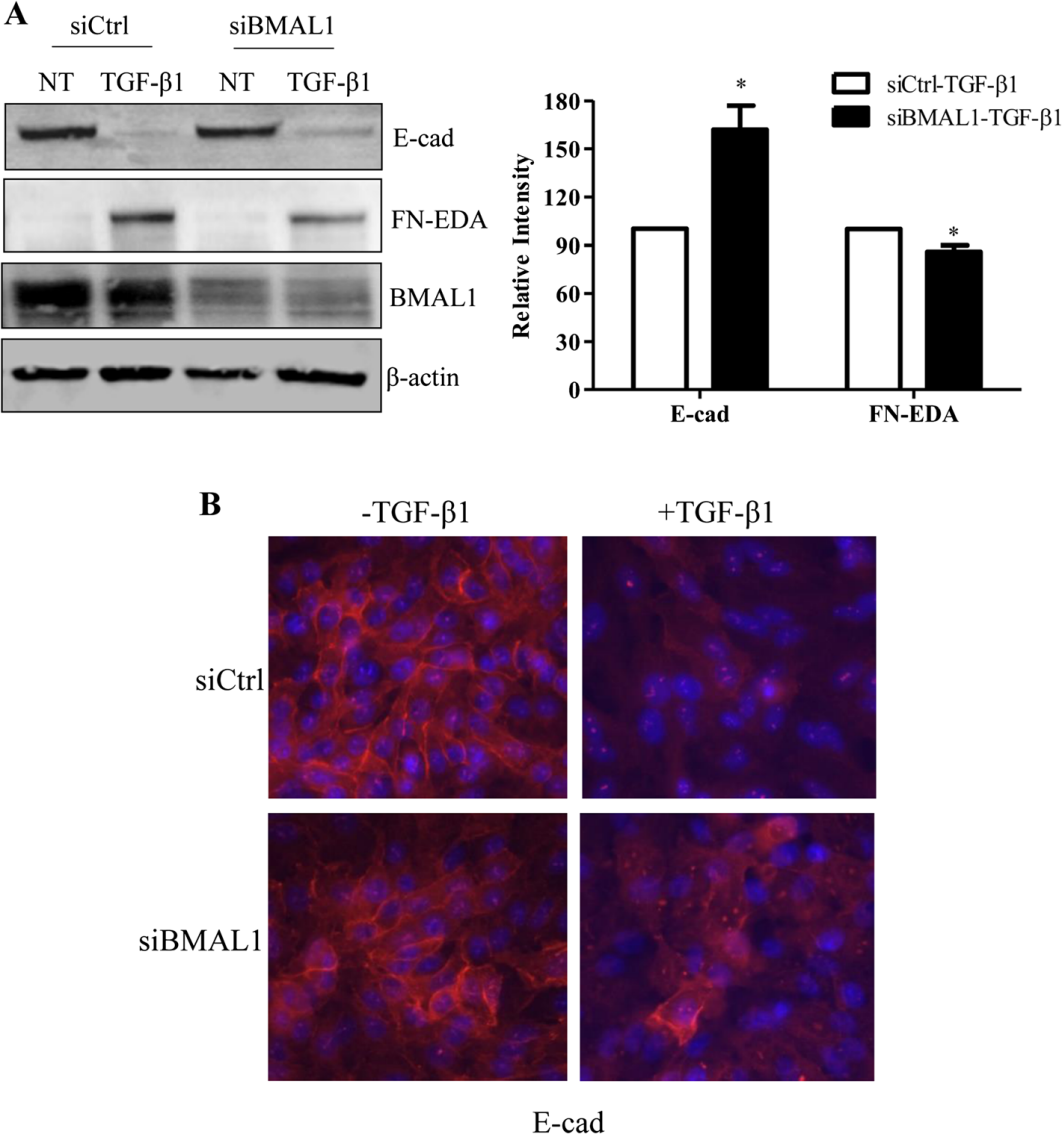
Effect of BMAL1 knockdown on TGF-β1-induced EMT of A549 human lung carcinoma cells. **a** A549 cells were transiently transfected with siBMAL1 or siCtrl for 72 h. During the last 48 h of transfection, the cells were treated with TGF-β1 at 5 ng/ml. **a** Western blot analysis was carried out to investigate the expression of EMT markers and BMAL1. * *p* < 0.05 vs. corresponding control siRNA groups stimulated with TGF-β1 (siCtrl-TGF-β1). **b** Immunofluorescent staining of E-cad in response to BMAL1 knockdown in A549 cells, 20× magnification, red, E-cad; Blue, DAPI

TGF-β1 promotes EMT of lung epithelial cells, which is one of the possible sources of myofibroblast accumulation in the fibrotic lung [42, 43]. siRNA against BMAL1 was transfected into HPL1D cells for 24 h, followed by TGF-β1 treatment at a concentration of 5 ng/ml for another 48 h. Real-time quantitative RT-PCR analysis showed that siRNA transfection effectively knocked down the expression of BMAL1 by 57 % in HPL1D cells (Fig. 5a). RT-PCR and Western blot analyses revealed that TGF-β1-induced loss of epithelial cell marker, E-cad was partially reversed by BMAL1 knockdown at both mRNA and protein levels (Fig. 5b, d, e). Meanwhile, the TGF-β1-induced expression of one of the mesenchymal markers, FN-EDA was significantly attenuated by BMAL1 knockdown (Fig. 5c, d, f). Similar results were obtained with A549 cells. BMAL1 knockdown partially reversed TGF-β1-induced loss of epithelial cell marker E-cad as revealed in Western blot analysis (Fig. 6a) and immunofluorescent staining (Fig. 6b) . In addition, up-regulation of FN-EDA, a mesenchymal marker, was revealed by Western blot analysis (Fig. 6a). Furthermore, using a TGF-β1 signaling target RT2 Profiler PCR Array, we determined that the expression of several other TGF-β1-induced genes including TGFBI, THBS1, PDGFB, TMEPA1 and JUNB, were also significantly attenuated in BMAL1-deficient cells (data not shown).

### siRNA-mediated knockdown of BMAL1 attenuated TGF-β1-induced-MMP9 production

**Fig. 7:**
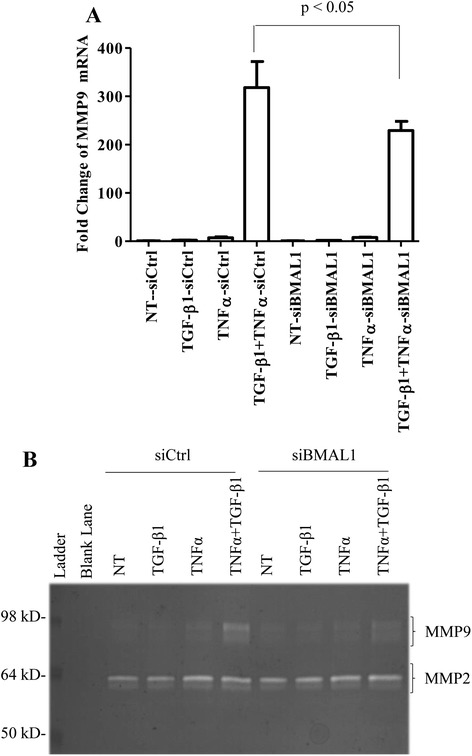
Effect of BMAL1 on MMP9 production. HPL1D cells were transiently transfected with siBMAL1 or siCtrl for 48 h. During the last 24 h of transfection, the cells were treated with TGF-β1 at 5 ng/ml and/or TNFα at 10 ng/ml. Cell pellets were harvested for RNA isolation and conditioned media were collected for zymography. **a** Real-time qRT-PCR analysis of MMP9 in HPL1D cells; **b** Gelatin zymographic analysis of MMP9 activity in HPL1D cells

TGF-β1 has been shown to induce the production of MMP9 in several different cell lines, and MMP9 level is elevated in lung fibrosis [44–48]. Our qRT-PCR and gelatin zymographic results demonstrated that TGF-β1 alone had no effect on the MMP9 production in HPL1D cells (Fig. 7a, b). However, when we treated HPL1D cells with TGF-β1 together with TNFα, the MMP9 levels increased as much as 300-fold at mRNA level as revealed by real-time qRT-PCR analysis (Fig. 7a). Meanwhile, knockdown of BMAL1 significantly attenuated the up-regulation of MMP9 induced by TGF-β1 and TNFα by 28 % (Fig. 7a). Both pro- and active MMP9 in cell culture medium were measured by zymographic analysis and the results showed the level of pro-MMP9, but not the active form of MMP9, was down-regulated by BMAL1 knockdown (Fig. 7b). However, BMAL1 did not affect the production of TGF-β1-induced MMP2 production (Fig. 7b).

### siRNA-mediated knockdown of BMAL1 attenuated TGF-β1-induced cell migration

**Fig. 8:**
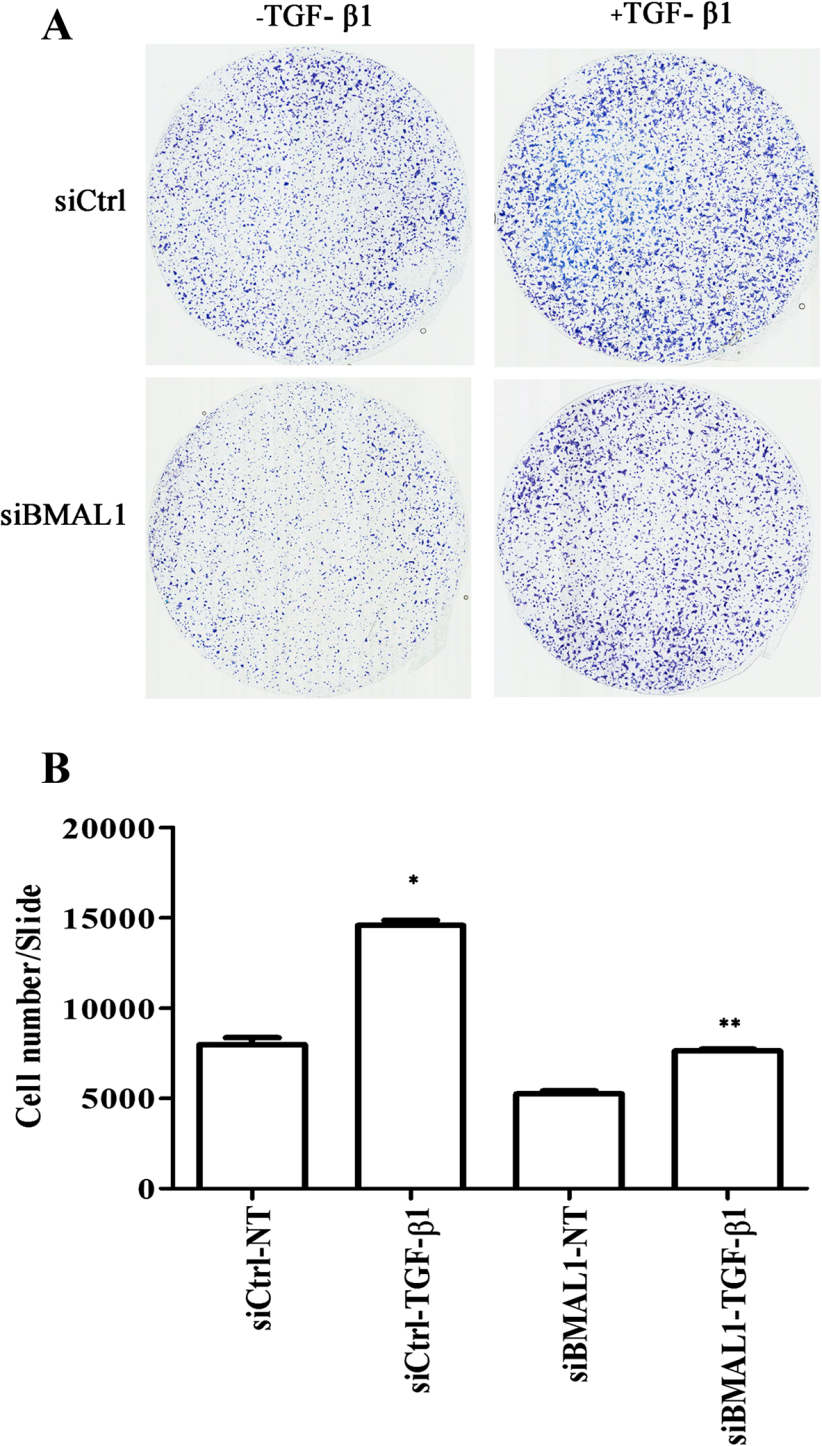
Effect of siBMAL1 on HPL1D cell migration. HPL1D cells in 6-well plates were transfected with siBMAL1 or siCtrl for 48 h and treated with TGF-β1 for 24 h. Equal numbers of cells were seeded in non-coated transwell inserts and cultured for another 24 h. Cells migrated through the membranes were stained and the membranes were mounted on slides with immersion oil. **a** Representative membranes showing cells migrated through transwells. **b** Quantification of numbers of cells migrated through membranes. The slides were scanned by slide scanner and the total cell numbers were counted with ImageJ software. * *p* < 0.05 *vs.* siCtrl-NT; ** *p* < 0.05 *vs.* siCtrl-TGF-β1

It is well known that cells that undergo EMT acquire increased migratory capabilities. As demonstrated above, BMAL1 is required for the TGF-β1-induced EMT and MMP9 production; we next examine whether the TGF-β1-induced epithelial cell migration requires BMAL1. siBMAL1 was transfected into HPL1D cells for 24 h followed by TGF-β1 treatment for another 48 h. Equal numbers of cells were then seeded in uncoated transwell inserts. F12 medium containing 5 % of FBS were added in the bottom chambers as chemoattractant. After incubation for 24 h, cells that passed through the membrane were stained with Diff-Quik solution following manufacturer’s instruction and the cell numbers were counted. As shown in Fig. 8, TGF-β1 promoted the migration of HPL1D cells by 2-fold which was markedly alleviated by BMAL1 knockdown.

### BMAL1 regulates Smad3 activation in alveolar epithelial cells

**Fig. 9:**
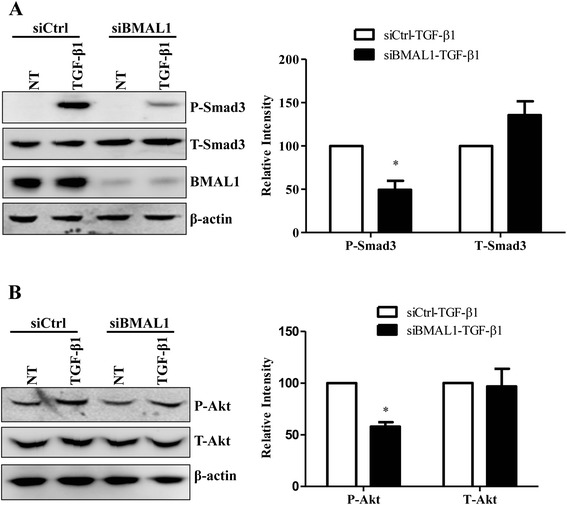
Effect of BMAL1 knockdown on TGF-β1-induced signaling transduction. **a** HPL1D cells in 6-well plates were transfected with siBMAL1 or siCtrl for 48 h and stimulated with TGF-β1 at 5 ng/ml for 30 min. The level of phosphorylated Smad3 was detected with phospho-specific antibodies. The total level of Smad3 was also investigated. **b** NHLFs in 6-well plates were transfected with siBMAL1 or siCtrl for 48 h and stimulated with TGF-β1 at 1 ng/ml for 18 h. The levels of phosphorylated and total Akt were detected with specific antibodies. * *p* < 0.05 vs. corresponding control siRNA groups stimulated with TGF-β1 (siCtrl-TGF-β1)

Smad2/3 signaling pathway is the canonical TGF-β1 signaling pathway responsible for the expression of a number of TGF-β1 target genes. We next examine the effect of BMAL1 on the Smad2/3 activation in lung epithelial cells. Following transfection siBMAL1 into the HPL1D cells for 3 days, cells were stimulated with TGF-β1 at 5 ng/ml for 30 min. The activation of Smad3 was measured by the western blot using phospho-specific antibodies. Our results showed phosphorylation of Smad3 was markedly attenuated in cells transfected with siBMAL1 in HPL1D lung epithelial cells (Fig. 9a). Meanwhile, the total expression levels of Smad3 was not affected in the cells transfected with siBMAL1 compared to the control siRNA (Fig. 9a).

Besides the classic Smad2/3 pathway, the Akt signaling pathway has been reported to be involved in the FMD of lung fibroblast cells [49]. We found that knockdown of BMAL1 decreased the TGF-β1-induced phosphorylation of Akt without affecting the total expression level of Akt (Fig. 9b).

### BMAL1 regulates Smad3 activity in a glycogen synthase kinase-3 beta (GSK3β) dependent manner

**Fig. 10:**
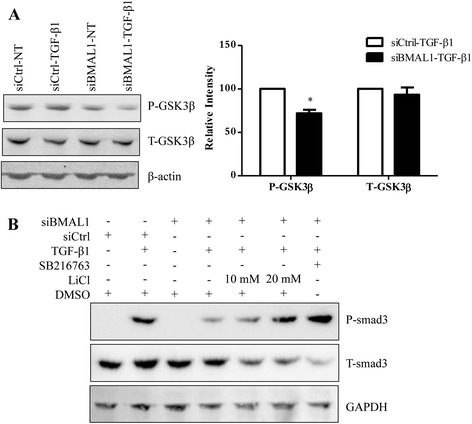
Involvement of GSK3β in the regulation of BMAL1 on TGF-β1-induced Smad3 activation. **a** A549 cells transfected with siBMAL1 or control siRNA for 48 h and treated with TGF-β1 for another 8 h. The levels of phosphorylated (at Ser9) and total GSK3β were detected with specific antibodies. * *p* < 0.05 vs. corresponding control siRNA groups stimulated with TGF-β1 (siCtrl-TGF-β1). **b** A549 cells transfected with siBMAL1 or siCtrl for 48 h were pretreated with GSK3β inhibitors, LiCl or SB216763, for two and a half hours and then treated with TGF-β1 for another 30 min. The levels of phosphorylated and total Smad3 were detected with specific antibodies

It has been reported that GSK3β interacts with Smad3 to negatively regulate Smad-mediated transcription responses [50]. Meanwhile, the phosphorylation of GSK3β at Ser9, which inactivates GSK3β [51] has been demonstrated to be subject to the regulation of circadian rhythm [52]. Therefore, we hypothesized that GSK3β mediates the regulation of TGF-β1-induced Smad3 activation by BMAL1. To test this hypothesis we first investigated whether BMAL1 is involved in the degradation of GSK3β in alveolar epithelial cells. Phospho-specific antibody reacting with phosphorylated Ser9 of GSK3β was employed to detect the inactive form of GSK3β. As shown in Fig. 10a, knockdown of BMAL1 in A549 cells dramatically down-regulated the levels of GSK3β (S9) without affecting its total level, suggesting that knockdown BMAL1 increases the level of active form of GSK3β.

In the next experiment, using inhibitors of GSK3β, we found that GSK3β inhibitors, LiCl and SB216763, were able to partially rescue the effects of BMAL1 knockdown on Smad3 phosphorylation, as seen in Western blot analysis (Fig. 10b). Therefore, the effect of BMAL1 on TGF-β1-induced Smad3 activation may be partially mediated through GSK3β, which needs further investigation.

## Discussion

Deregulation of circadian clock genes have been implicated in loss of cell cycle control, impaired DNA damage repair, and tumor formation [53]. The circadian clock also regulates the NRF2/glutathione-mediated antioxidant defense in the mouse lung [54]. Our in vivo studies demonstrated that TGF-β1 alters the expression of the clock genes, named *BMAL1, NPAS2, Per1*, *Per2*, *Per3*, *Rev-erbα*, *RORα* and *DBP.* Nevertheless, to determine the full effects of TGF-β1 in the circadian clock regulation in vivo, further studies will be necessary using mice exposed to different light/dark time, and analysis every 4 h, during the course of 24–36 h. This study focused in the specific role of the transcription factor BMAL1 in the process of fibrogenesis, taking in count that BMAL1 occupancy is known in more than 150 sites, including genes that encode central regulators of metabolic and rhythmic processes [55]. In addition, BMAL1 has also been reported to be involved in cell proliferation and cancer cell invasion through regulating different signaling pathways [56–58]. Here, we demonstrated that BMAL1 participates in the process of myofibroblast differentiation mediated by TGF-β1. We propose that the cell-intrinsic clock machinery and the expression of specific clock genes, such as BMAL1, could be crucial in the understanding of the biology of myofibroblast, a key player in pulmonary fibrosis as well as tumor progression.

TGF-β1 dependent signaling and metabolic reprogramming are essential components of EMT, metastasis and tissue fibrosis [59, 60]. Interestingly, BMAL1 binds both *Hif1*α and *Vegfa* to directly control specific pathways such as glucose metabolism and triglyceride metabolism [61]. BMAL1 also plays an important role in cellular differentiation, as shown in studies with brown adipocytes, through direct transcriptional control of key components of the TGF-β pathway [39]. Furthermore, promoter analysis in other cell types revealed that TGF-β regulators such as *Smad7*, *Lefty*, *Smurf2, Smad9*, *Itga6* as well as modulators of Bmp and Notch signaling contain putative Bmal1/Clock-binding sites within their proximal and distal promoter regions [23]. The present study demonstrated that abrogation of BMAL1 prevents the mesenchymal-like morphology induced by TGF-β1, confirming that inhibition of BMAL1 abrogates TGF-β1-induced gene expression and myofibroblast differentiation. Our findings indicate that inhibition of BMAL1 expression prevents not only EMT but also FMD, two major differentiation processes involved in the pathophysiology of pulmonary fibrosis as well as stromal support during tumor progression. We demonstrated that TGF-β1-induced expression of PAI1, which is overexpressed in pulmonary fibrosis and lung carcinoma, is inhibited upon inactivation of BMAL1 in alveolar epithelial cells as well as lung fibroblasts. Previous studies mechanistically support those findings, as site-directed mutagenesis studies suggest that heterodimers BMAL1/CLOCK act on two canonical E-boxes to regulate PAI1 promoter activity [62].

Studies on the suprachiasmatic nucleus revealed that GSK3β pathway regulates circadian gene expression by controlling BMAL1 protein stability in vivo [52]. GSK3β specifically phosphorylates BMAL1 and primes it for ubiquitination, followed by proteasomal degradation [63]. Our study of lung epithelial cells revealed that GSK3β phosphorylation (inactivation) is reduced upon down-regulation of BMAL1 expression, indicating that BMAL1 regulates GSK3β degradation and that BMAL1 and GSK3β mutually control each other.

Mechanistically, GSK3β inhibits profibrotic transforming growth factor-β1/Smad3 signaling, probably via interaction with Smad3, as reported in cardiac fibroblasts. Deletion of GSK3β or inactivation can increase Smad3 transcriptional activity [64]. Active GSK3β mediates phosphorylation of Smad3, promoting Smad3 ubiquitination and inhibition the Smad3 transcriptional activity [64, 65]. Our results showed that inhibition of Smad3 activation by BMAL1 knockdown was partially reversed by GSK3β inhibitors. Therefore, we proposed that BMAL1 contributes to the TGF-β1 induced Smad3 activation through a mechanism that involves the regulation of GSK3β.

TGF-β1/Smad3 signaling pathway has critical functional roles in the development of both emphysema and fibrosis in the lung [66–70]. For example, null mutation of Smad3 protects mice against fibrosis induced by bleomycin [70]. Disruption of TGF-β1 signaling by Smad3 inactivation has also been shown to promote emphysema and resistance to pulmonary fibrosis [69]. In fact, recent studies revealed that targeted deletion of BMAL1 in lung epithelium promotes inflammation and emphysema [34]. Nevertheless, the susceptibility to pulmonary fibrosis has not been established. Our results suggest that BMAL1 could play a key role in determining the type of response to lung injury. A recent study revealed that BMAL1 is a substrate of S6K1, implicating BMAL1 as a translational factor [71]. Those studies linked for the first time the circadian clock with the mTOR signaling pathway, a pathway known to be altered by TGF-β1 in pulmonary fibrosis [36, 71]. From the translational point of view, it is important to recognize that clock-amplitude enhancing small molecules can help to retard or even reverse some of the physiological decline and improve metabolic and physiological well-being [72]. Then, direct regulators of the circadian clock genes could contribute to modulate the progression of lung cancer and pulmonary fibrosis. For example, melatonin, a pineal hormone that helps maintain circadian rhythm, was reported to prevent pulmonary fibrosis mediated by bleomycin and endoplasmic reticulum stress in animal models [73, 74]. Melatonin also inhibits the proliferation of the pro-fibrotic fibroblasts derived from scleroderma patients and the migration of human lung adenocarcinoma A549 cell lines [75–79]. Mechanistically, melatonin blocks the expression of the clock gene *BMAL1*, via repression of RORα transcriptional activity [80]. Furthermore, Melatonin’s blockade of BMAL1 expression is associated with the decreased expression of Sirtuin 1 (SIRT1), a nutrient sensing deacetylase, which interacts with CLOCK to regulate BMAL1 acetylation [81, 82]. Interestingly, knockdown of SIRT1 can effectively inhibit TGF-β1 signaling and exerts potent antifibrotic effects, establishing SIRT1 as a key regulator of fibroblast activation in systemic sclerosis [83]. Those findings suggest a regulatory pathway against fibrogenesis that is Melatonin/Sirt1/Bmal1 dependent.

## Conclusions

In conclusion, we demonstrated that activation of TGF-β1 promotes the transcriptional induction of BMAL1. TGF-β1 promotes high levels of deacetylated BMAL1, probably through a SIRT1-dependent mechanism. And finally, BMAL1 expression induced by TGF-β1 in the lung has a profibrotic role. Nevertheless, the pathways involved in the crosstalk between TGF-β1 signaling and the circadian clock still need further investigation as well as the translational relevance in cancer and fibrogenesis, considering the notion of a relationship between sleep disruption, aging, obesity and cancer [84, 85]. With the future development of clock regulating small molecules, pharmacological agents targeting sleep and circadian clocks promise future applications in age and metabolic related lung diseases.

## References

[1] Selman M, Pardo A (2012). Alveolar epithelial cell disintegrity and subsequent activation: a key process in pulmonary fibrosis. Am J Respir Crit Care Med.

[2] Allen JT, Spiteri MA (2002). Growth factors in idiopathic pulmonary fibrosis: relative roles. Respir Res.

[3] Selman M, Pardo A (2014). Revealing the pathogenic and aging-related mechanisms of the enigmatic idiopathic pulmonary fibrosis. an integral model. Am J Respir Crit Care Med.

[4] King TE, Pardo A, Selman M (2011). Idiopathic pulmonary fibrosis. Lancet.

[5] Yue X, Shan B, Lasky JA. TGF-β: Titan of Lung Fibrogenesis.Curr Enzym Inhib. 2010 Jul 1;6(2). doi: 10.2174/10067. 10.2174/10067 PMC3812949 24187529

[6] Hoyt DG, Lazo JS (1988). Alterations in pulmonary mRNA encoding procollagens, fibronectin and transforming growth factor-beta precede bleomycin-induced pulmonary fibrosis in mice. J Pharmacol Exp Ther.

[7] Phan SH, Kunkel SL (1992). Lung cytokine production in bleomycin-induced pulmonary fibrosis. Exp Lung Res.

[8] Yi ES, Bedoya A, Lee H, Chin E, Saunders W, Kim SJ (1996). Radiation-induced lung injury in vivo: expression of transforming growth factor-beta precedes fibrosis. Inflammation.

[9] Sime PJ, Xing Z, Graham FL, Csaky KG, Gauldie J (1997). Adenovector-mediated gene transfer of active transforming growth factor-beta1 induces prolonged severe fibrosis in rat lung. J Clin Invest.

[10] Warshamana GS, Pociask DA, Fisher KJ, Liu JY, Sime PJ, Brody AR (2002). Titration of non-replicating adenovirus as a vector for transducing active TGF-beta1 gene expression causing inflammation and fibrogenesis in the lungs of C57BL/6 mice. Int J Exp Pathol.

[11] Puthawala K, Hadjiangelis N, Jacoby SC, Bayongan E, Zhao Z, Yang Z (2008). Inhibition of integrin alpha(v)beta6, an activator of latent transforming growth factor-beta, prevents radiation-induced lung fibrosis. Am J Respir Crit Care Med.

[12] Giri SN, Hyde DM, Hollinger MA (1993). Effect of antibody to transforming growth factor beta on bleomycin induced accumulation of lung collagen in mice. Thorax.

[13] Huang F, Chen YG (2012). Regulation of TGF-beta receptor activity. Cell Biosci.

[14] Moustakas A, Heldin CH (2009). The regulation of TGFbeta signal transduction. Development.

[15] Derynck R, Zhang YE (2003). Smad-dependent and Smad-independent pathways in TGF-beta family signalling. Nature.

[16] Moustakas A, Heldin CH (2005). Non-Smad TGF-beta signals. J Cell Sci.

[17] Zhang YE (2009). Non-Smad pathways in TGF-beta signaling. Cell Res.

[18] Ko CH, Takahashi JS (2006). Molecular components of the mammalian circadian clock. Hum Mol Genet.

[19] Gekakis N, Staknis D, Nguyen HB, Davis FC, Wilsbacher LD, King DP (1998). Role of the CLOCK protein in the mammalian circadian mechanism. Science.

[20] Takahashi JS, Hong HK, Ko CH, McDearmon EL (2008). The genetics of mammalian circadian order and disorder: implications for physiology and disease. Nat Rev Genet.

[21] Bunger MK, Wilsbacher LD, Moran SM, Clendenin C, Radcliffe LA, Hogenesch JB (2000). Mop3 is an essential component of the master circadian pacemaker in mammals. Cell.

[22] Kondratov RV, Kondratova AA, Gorbacheva VY, Vykhovanets OV, Antoch MP (2006). Early aging and age-related pathologies in mice deficient in BMAL1, the core componentof the circadian clock. Genes Dev.

[23] Janich P, Pascual G, Merlos-Suarez A, Batlle E, Ripperger J, Albrecht U (2011). The circadian molecular clock creates epidermal stem cell heterogeneity. Nature.

[24] Woon PY, Kaisaki PJ, Braganca J, Bihoreau MT, Levy JC, Farrall M (2007). Aryl hydrocarbon receptor nuclear translocator-like (BMAL1) is associated with susceptibility to hypertension and type 2 diabetes. Proc Natl Acad Sci U S A.

[25] Cho H, Zhao X, Hatori M, Yu RT, Barish GD, Lam MT (2012). Regulation of circadian behaviour and metabolism by REV-ERB-alpha and REV-ERB-beta. Nature.

[26] Akashi M, Takumi T (2005). The orphan nuclear receptor RORalpha regulates circadian transcription of the mammalian core-clock Bmal1. Nat Struct Mol Biol.

[27] Gibbs J, Ince L, Matthews L, Mei J, Bell T, Yang N (2014). An epithelial circadian clock controls pulmonary inflammation and glucocorticoid action. Nat Med.

[28] Sun X, Dang F, Zhang D, Yuan Y, Zhang C, Wu Y (2015). Glucagon-CREB/CRTC2 signaling cascade regulates hepatic BMAL1 protein. J Biol Chem.

[29] Hiragaki S, Baba K, Coulson E, Kunst S, Spessert R, Tosini G (2014). Melatonin signaling modulates clock genes expression in the mouse retina. PLoS One.

[30] Cavadini G, Petrzilka S, Kohler P, Jud C, Tobler I, Birchler T (2007). TNF-alpha suppresses the expression of clock genes by interfering with E-box-mediated transcription. Proc Natl Acad Sci U S A.

[31] Gast H, Gordic S, Petrzilka S, Lopez M, Muller A, Gietl A (2012). Transforming growth factor-beta inhibits the expression of clock genes. Ann N Y Acad Sci.

[32] Gibbs JE, Beesley S, Plumb J, Singh D, Farrow S, Ray DW (2009). Circadian timing in the lung; a specific role for bronchiolar epithelial cells. Endocrinology.

[33] Hadden H, Soldin SJ, Massaro D (2012). Circadian disruption alters mouse lung clock gene expression and lung mechanics. J Appl Physiol (1985).

[34] Hwang JW, Sundar IK, Yao H, Sellix MT, Rahman I (2014). Circadian clock function is disrupted by environmental tobacco/cigarette smoke, leading to lung inflammation and injury via a SIRT1-BMAL1 pathway. FASEB J.

[35] Masuda A, Kondo M, Saito T, Yatabe Y, Kobayashi T, Okamoto M (1997). Establishment of human peripheral lung epithelial cell lines (HPL1) retaining differentiated characteristics and responsiveness to epidermal growth factor, hepatocyte growth factor, and transforming growth factor beta1. Cancer Res.

[36] Sosulski ML, Gongora R, Danchuk S, Dong C, Luo F, Sanchez CG (2015). Deregulation of selective autophagy during aging and pulmonary fibrosis: the role of TGFbeta1. Aging Cell..

[37] Becerril C, Pardo A, Montano M, Ramos C, Ramirez R, Selman M (1999). Acidic fibroblast growth factor induces an antifibrogenic phenotype in human lung fibroblasts. Am J Respir Cell Mol Biol.

[38] Abdalla M, Sabbineni H, Prakash R, Ergul A, Fagan SC, Somanath PR (2015). The Akt inhibitor, triciribine, ameliorates chronic hypoxia-induced vascular pruning and TGFbeta-induced pulmonary fibrosis. Br J Pharmacol.

[39] Nam D, Guo B, Chatterjee S, Chen MH, Nelson D, Yechoor VK (2015). The adipocyte clock controls brown adipogenesis through the TGF-beta and BMP signaling pathways. J Cell Sci.

[40] Kondratov RV, Chernov MV, Kondratova AA, Gorbacheva VY, Gudkov AV, Antoch MP (2003). BMAL1-dependent circadian oscillation of nuclear CLOCK: posttranslational events induced by dimerization of transcriptional activators of the mammalian clock system. Genes Dev.

[41] Huang TS, Grodeland G, Sleire L, Wang MY, Kvalheim G, Laerum OD (2009). Induction of circadian rhythm in cultured human mesenchymal stem cells by serum shock and cAMP analogs in vitro. Chronobiol Int.

[42] Willis BC, Borok Z (2006). Epithelial origin of myofibroblasts during fibrosis in the lung. Proc Am Thorac Soc.

[43] Kasai H, Allen JT, Mason RM, Kamimura T, Zhang Z (2005). TGF-beta1 induces human alveolar epithelial to mesenchymal cell transition (EMT). Respir Res.

[44] Kim HS, Shang T, Chen Z, Pflugfelder SC, Li DQ (2004). TGF-beta1 stimulates production of gelatinase (MMP-9), collagenases (MMP-1, −13) and stromelysins (MMP-3, −10, −11) by human corneal epithelial cells. Exp Eye Res.

[45] Sinpitaksakul SN, Pimkhaokham A, Sanchavanakit N, Pavasant P (2008). TGF-beta1 induced MMP-9 expression in HNSCC cell lines via Smad/MLCK pathway. Biochem Biophys Res Commun.

[46] Gomes LR, Terra LF, Wailemann RA, Labriola L, Sogayar MC (2012). TGF-beta1 modulates the homeostasis between MMPs and MMP inhibitors through p38 MAPK and ERK1/2 in highly invasive breast cancer cells. BMC Cancer.

[47] Selman M, Ruiz V, Cabrera S, Segura L, Ramirez R, Barrios R (2000). TIMP-1, −2, −3, and −4 in idiopathic pulmonary fibrosis. A prevailing nondegradative lung microenvironment?. Am J Physiol Lung Cell Mol Physiol.

[48] Pardo A, Selman M (2012). Role of matrix metaloproteases in idiopathic pulmonary fibrosis. Fibrogenesis Tissue Repair.

[49] Guo W, Shan B, Klingsberg RC, Qin X, Lasky JA (2009). Abrogation of TGF-beta1-induced fibroblast-myofibroblast differentiation by histone deacetylase inhibition. Am J Physiol Lung Cell Mol Physiol.

[50] Hua F, Zhou J, Liu J, Zhu C, Cui B, Lin H (2010). Glycogen synthase kinase-3beta negatively regulates TGF-beta1 and Angiotensin II-mediated cellular activity through interaction with Smad3. Eur J Pharmacol.

[51] Lamarre M, Desrosiers RR (2008). Up-regulation of protein L-isoaspartyl methyltransferase expression by lithium is mediated by glycogen synthase kinase-3 inactivation and beta-catenin stabilization. Neuropharmacology.

[52] Besing RC, Paul JR, Hablitz LM, Rogers CO, Johnson RL, Young ME (2015). Circadian rhythmicity of active GSK3 isoforms modulates molecular clock gene rhythms in the suprachiasmatic nucleus. J Biol Rhythms.

[53] Yu EA, Weaver DR (2011). Disrupting the circadian clock: gene-specific effects on aging, cancer, and other phenotypes. Aging (Albany NY).

[54] Pekovic-Vaughan V, Gibbs J, Yoshitane H, Yang N, Pathiranage D, Guo B (2014). The circadian clock regulates rhythmic activation of the NRF2/glutathione-mediated antioxidant defense pathway to modulate pulmonary fibrosis. Genes Dev.

[55] Hatanaka F, Matsubara C, Myung J, Yoritaka T, Kamimura N, Tsutsumi S (2010). Genome-wide profiling of the core clock protein BMAL1 targets reveals a strict relationship with metabolism. Mol Cell Biol.

[56] McDearmon EL, Patel KN, Ko CH, Walisser JA, Schook AC, Chong JL (2006). Dissecting the functions of the mammalian clock protein BMAL1 by tissue-specific rescue in mice. Science.

[57] Lin F, Chen Y, Li X, Zhao Q, Tan Z (2013). Over-expression of circadian clock gene Bmal1 affects proliferation and the canonical Wnt pathway in NIH-3 T3 cells. Cell Biochem Funct.

[58] Jung CH, Kim EM, Park JK, Hwang SG, Moon SK, Kim WJ (2013). Bmal1 suppresses cancer cell invasion by blocking the phosphoinositide 3-kinase-Akt-MMP-2 signaling pathway. Oncol Rep.

[59] Jiang L, Xiao L, Sugiura H, Huang X, Ali A, Kuro OM (2015). Metabolic reprogramming during TGFbeta1-induced epithelial-to-mesenchymal transition. Oncogene..

[60] Tsukamoto H (2015). Metabolic reprogramming and cell fate regulation in alcoholic liver disease. Pancreatology..

[61] Rey G, Cesbron F, Rougemont J, Reinke H, Brunner M, Naef F (2011). Genome-wide and phase-specific DNA-binding rhythms of BMAL1 control circadian output functions in mouse liver. PLoS Biol.

[62] Chong NW, Codd V, Chan D, Samani NJ (2006). Circadian clock genes cause activation of the human PAI-1 gene promoter with 4G/5G allelic preference. FEBS Lett.

[63] Sahar S, Zocchi L, Kinoshita C, Borrelli E, Sassone-Corsi P (2010). Regulation of BMAL1 protein stability and circadian function by GSK3beta-mediated phosphorylation. PLoS One.

[64] Lal H, Ahmad F, Zhou J, Yu JE, Vagnozzi RJ, Guo Y (2014). Cardiac fibroblast glycogen synthase kinase-3beta regulates ventricular remodeling and dysfunction in ischemic heart. Circulation.

[65] Guo X, Ramirez A, Waddell DS, Li Z, Liu X, Wang XF (2008). Axin and GSK3- control Smad3 protein stability and modulate TGF- signaling. Genes Dev.

[66] Warburton D, Shi W, Xu B (2013). TGF-beta-Smad3 signaling in emphysema and pulmonary fibrosis: an epigenetic aberration of normal development?. Am J Physiol Lung Cell Mol Physiol.

[67] Li M, Krishnaveni MS, Li C, Zhou B, Xing Y, Banfalvi A (2011). Epithelium-specific deletion of TGF-beta receptor type II protects mice from bleomycin-induced pulmonary fibrosis. J Clin Invest.

[68] Gauldie J, Kolb M, Ask K, Martin G, Bonniaud P, Warburton D (2006). Smad3 signaling involved in pulmonary fibrosis and emphysema. Proc Am Thorac Soc.

[69] Bonniaud P, Kolb M, Galt T, Robertson J, Robbins C, Stampfli M (2004). Smad3 null mice develop airspace enlargement and are resistant to TGF-beta-mediated pulmonary fibrosis. J Immunol.

[70] Zhao J, Shi W, Wang YL, Chen H, Bringas P, Datto MB (2002). Smad3 deficiency attenuates bleomycin-induced pulmonary fibrosis in mice. Am J Physiol Lung Cell Mol Physiol.

[71] Lipton JO, Yuan ED, Boyle LM, Ebrahimi-Fakhari D, Kwiatkowski E, Nathan A (2015). The circadian protein BMAL1 regulates translation in response to S6K1-mediated phosphorylation. Cell.

[72] Nohara K, Yoo SH, Chen ZJ (2015). Manipulating the circadian and sleep cycles to protect against metabolic disease. Front Endocrinol (Lausanne).

[73] Zhao H, Wu QQ, Cao LF, Qing HY, Zhang C, Chen YH (2014). Melatonin inhibits endoplasmic reticulum stress and epithelial-mesenchymal transition during bleomycin-induced pulmonary fibrosis in mice. PLoS One.

[74] Arslan SO, Zerin M, Vural H, Coskun A (2002). The effect of melatonin on bleomycin-induced pulmonary fibrosis in rats. J Pineal Res.

[75] Todisco M (2006). Effectiveness of a treatment based on melatonin in five patients with systemic sclerosis. Am J Ther.

[76] Simonini G, Pignone A, Generini S, Falcini F, Cerinic MM (2000). Emerging potentials for an antioxidant therapy as a new approach to the treatment of systemic sclerosis. Toxicology.

[77] Carossino AM, Lombardi A, Matucci-Cerinic M, Pignone A, Cagnoni M (1996). Effect of melatonin on normal and sclerodermic skin fibroblast proliferation. Clin Exp Rheumatol.

[78] Lee YJ, Lee JH, Moon JH, Park SY (2014). Overcoming hypoxic-resistance of tumor cells to TRAIL-induced apoptosis through melatonin. Int J Mol Sci.

[79] Zhou Q, Gui S, Zhou Q, Wang Y (2014). Melatonin inhibits the migration of human lung adenocarcinoma A549 cell lines involving JNK/MAPK pathway. PLoS One.

[80] Hill SM, Frasch T, Xiang S, Yuan L, Duplessis T, Mao L (2009). Molecular mechanisms of melatonin anticancer effects. Integr Cancer Ther.

[81] Jung-Hynes B, Reiter RJ, Ahmad N (2010). Sirtuins, melatonin and circadian rhythms: building a bridge between aging and cancer. J Pineal Res.

[82] Nakahata Y, Kaluzova M, Grimaldi B, Sahar S, Hirayama J, Chen D (2008). The NAD + −dependent deacetylase SIRT1 modulates CLOCK-mediated chromatin remodeling and circadian control. Cell.

[83] Zerr P, Palumbo-Zerr K, Huang J, Tomcik M, Sumova B, Distler O (2016). Sirt1 regulates canonical TGFbeta signalling to control fibroblast activation and tissue fibrosis. Ann Rheum Dis..

[84] Haus EL, Smolensky MH (2013). Shift work and cancer risk: potential mechanistic roles of circadian disruption, light at night, and sleep deprivation. Sleep Med Rev.

[85] Narasimamurthy R, Hatori M, Nayak SK, Liu F, Panda S, Verma IM (2012). Circadian clock protein cryptochrome regulates the expression of proinflammatory cytokines. Proc Natl Acad Sci U S A.

